# Dopamine Increases CD14^+^CD16^+^ Monocyte Migration and Adhesion in the Context of Substance Abuse and HIV Neuropathogenesis

**DOI:** 10.1371/journal.pone.0117450

**Published:** 2015-02-03

**Authors:** Jacqueline S. Coley, Tina M. Calderon, Peter J. Gaskill, Eliseo A. Eugenin, Joan W. Berman

**Affiliations:** 1 Department of Pathology, Albert Einstein College of Medicine, Bronx, New York, United States of America; 2 Department of Microbiology and Immunology, Albert Einstein College of Medicine, Bronx, New York, United States of America; 3 Public Health Research Institute, New Jersey Medical School, Rutgers University, Newark, New Jersey, United States of America; University of Kentucky Medical Center, UNITED STATES

## Abstract

Drug abuse is a major comorbidity of HIV infection and cognitive disorders are often more severe in the drug abusing HIV infected population. CD14^+^CD16^+^ monocytes, a mature subpopulation of peripheral blood monocytes, are key mediators of HIV neuropathogenesis. Infected CD14^+^CD16^+^ monocyte transmigration across the blood brain barrier mediates HIV entry into the brain and establishes a viral reservoir within the CNS. Despite successful antiretroviral therapy, continued influx of CD14^+^CD16^+^ monocytes, both infected and uninfected, contributes to chronic neuroinflammation and the development of HIV associated neurocognitive disorders (HAND). Drug abuse increases extracellular dopamine in the CNS. Once in the brain, CD14^+^CD16^+^ monocytes can be exposed to extracellular dopamine due to drug abuse. The direct effects of dopamine on CD14^+^CD16^+^ monocytes and their contribution to HIV neuropathogenesis are not known. In this study, we showed that CD14^+^CD16^+^ monocytes express mRNA for all five dopamine receptors by qRT-PCR and D1R, D5R and D4R surface protein by flow cytometry. Dopamine and the D1-like dopamine receptor agonist, SKF38393, increased CD14^+^CD16^+^ monocyte migration that was characterized as chemokinesis. To determine whether dopamine affected cell motility and adhesion, live cell imaging was used to monitor the accumulation of CD14^+^CD16^+^ monocytes on the surface of a tissue culture dish. Dopamine increased the number and the rate at which CD14^+^CD16^+^ monocytes in suspension settled to the dish surface. In a spreading assay, dopamine increased the area of CD14^+^CD16^+^ monocytes during the early stages of cell adhesion. In addition, adhesion assays showed that the overall total number of adherent CD14^+^CD16^+^ monocytes increased in the presence of dopamine. These data suggest that elevated extracellular dopamine in the CNS of HIV infected drug abusers contributes to HIV neuropathogenesis by increasing the accumulation of CD14^+^CD16^+^ monocytes in dopamine rich brain regions.

## Introduction

HIV enters the brain within two weeks of peripheral infection [1,2]. The chronic, low level neuroinflammation that develops as a result of HIV infection of the central nervous system (CNS) is believed to lead to HIV associated neurocognitive disorders (HAND) in 40–70% of infected people, despite the success of combined antiretroviral therapy (cART) in reducing viral load in plasma and cerebrospinal fluid [3–5]. Monocyte transmigration across the blood brain barrier is an important mediator of HAND, as these cells bring HIV into the brain, initiating and propagating the neuroinflammation that can lead to cognitive impairment [6,7]. HIV infected individuals have increased CNS CXCL12 and CCL2 as a result of infection and activation of resident cells [8,9]. These chemokines can recruit infected and uninfected monocytes into the brain, further contributing to chronic neuroinflammation [8,10–14].

Monocyte maturation is critical to HIV neuropathogenesis [15,16]. Monocytes are broadly grouped according to expression of surface CD14 and CD16, the LPS and FcγRIII receptors, respectively. CD14^+^CD16^-^ monocytes constitute the majority of circulating monocytes. A small subset of monocytes expressing both CD14 and CD16 (CD14^+^CD16^+^) comprise 5–10% of total monocytes in healthy people and these cells are more mature than CD14^+^CD16^-^ monocytes [17,18]. The percentage of peripheral monocytes that are CD14^+^CD16^+^ is increased in HIV infected individuals [7,19,20]. Even in people on cART, this population remains increased [21]. The mature CD14^+^CD16^+^ monocyte population preferentially transmigrates across an in vitro human blood brain barrier model in response to CCL2 and is highly permissive to HIV infection [22–24]. During the neuropathogenesis of HIV and SIV, the nonhuman primate model of HIV, peripheral uninfected and infected CD14^+^CD16^+^ monocytes accumulate within the CNS [25–29]. Thus, uninfected and HIV infected CD14^+^CD16^+^ monocyte influx into the CNS contributes to neuroinflammation, CNS infection and establishment of viral reservoirs, and the development of HAND.

Drug abuse is a common comorbidity of HIV infection [30,31], and all drugs of abuse increase extracellular CNS dopamine, a neurotransmitter important for locomotion, cognition, and reward [32–37]. While there have been some studies of the effects of drug abuse on HIV associated neuroinflammation and HAND [38–42], little is known about the contribution of dopamine to HIV neuropathogenesis. In SIV infected macaques with increased CNS dopamine, there is more virus in dopamine rich brain regions and increased neuropathology [43,44]. These studies suggest that elevated extracellular dopamine in the CNS of HIV infected individuals abusing drugs increases neuroinflammation and exacerbates CNS disease. However, the mechanism(s) by which dopamine increases HIV associated neuroinflammation has not been extensively characterized.

Dopamine signals through G-protein coupled dopamine receptors that are grouped into two families. The D1-like dopamine receptors are D1R and D5R, and the D2-like dopamine receptors are D2R, D3R, and D4R. D1-like dopamine receptors signal through G_αs_ and stimulate adenylyl cyclase, while D2-like dopamine receptors signal through G_αi_ and inhibit adenylyl cyclase [45–47]. Monocytes differentiate into macrophages upon entering tissue, and our laboratory previously demonstrated dopamine receptor expression and function on human monocyte derived macrophages [48]. However, the expression of functional dopamine receptors by CD14^+^CD16^+^ monocytes is unknown.

Infiltrating monocytes, including the mature CD14^+^CD16^+^ monocyte subpopulation, are key mediators of HIV associated neuroinflammation and enter the CNS in response to increased CCL2 or CXCL12 [8–14,21]. Dopamine does not cross the BBB [49], but once within the brain, monocytes may encounter increased extrasynaptic dopamine due to acute and intermittent drug abuse [50,51]. Dopamine may affect the localization of CD14^+^CD16^+^ monocytes within the brain, increasing neuroinflammation in proximity of dopaminergic neurons. Therefore, we examined the direct effects of dopamine on CD14^+^CD16^+^ monocyte motility and accumulation, modeling dopamine mediated effects on these cells once within the CNS parenchyma of drug abusers. We found dopamine receptor mRNA and protein in freshly isolated monocytes, showed that dopamine receptor expression changes with monocyte maturation, and demonstrated that dopamine receptors on mature CD14^+^CD16^+^ monocytes are functional. We also showed that dopamine increases CD14^+^CD16^+^ monocyte migration, which is not gradient dependent. We demonstrated that the effects of dopamine on monocyte migration are mediated, at least in part, by D1-like dopamine receptor activation. Using live cell imaging, we demonstrated that dopamine increases the rate and number of CD14^+^CD16^+^ monocytes in suspension that settled to the surface of a tissue culture dish. In a spreading assay, dopamine increased the area of CD14^+^CD16^+^ monocytes during the early stages of adhesion. In addition, dopamine increased the overall number of adherent CD14^+^CD16^+^ monocytes in an adhesion assay. Our data indicate that elevated CNS dopamine due to drug abuse may increase the adhesion and accumulation of CD14^+^CD16^+^ monocytes in dopaminergic regions of the brain, contributing to HIV associated neuroinflammation.

## Materials and Methods

### Cell Isolation and Culture

Leukopaks were obtained from the New York Blood Center (New York City, NY) according to established protocols at the Albert Einstein College of Medicine. Peripheral blood mononuclear cells (PBMC) were isolated by Ficoll-Paque PLUS (GE Healthcare Life Sciences, Pittsburg, PA) density centrifugation, and monocytes were isolated from PBMC by magnetic separation using the EasySep Human CD14 Positive Selection Kit according to the manufacturer’s instructions (Stemcell Technologies, Vancouver, BC, Canada). CD14^+^ monocytes were resuspended in monocyte media (RPMI 1640 supplemented with 10% human serum, 5% FBS, 1% Pen/Strep, and 1% HEPES). Freshly isolated monocytes are termed “Day 0 monocytes.” Day 0 monocytes, which are 90–95% CD14^+^CD16^-^, were cultured non adherently at 2x10^6^ cells/mL with 10 ng/mL M-CSF in Teflon flasks for three days to yield mature/activated “Day 3 monocytes” that are enriched for CD14^+^CD16^+^ cells, as previously described [21,23].

### RNA isolation and qRT-PCR

Total RNA was isolated from Day 0 and Day 3 monocytes by Trizol (Life Technologies, Carlsbad, CA) extraction according to the manufacturer’s instructions and quantified by Nanodrop (Thermo Scientific, Wilmington, DE). Synthesis of cDNA was performed using a SuperScript VILO cDNA Synthesis Kit according to the manufacturer’s instructions (Life Technologies).

Relative mRNA expression of D1R, D2R, D3R, D4R, D5R, and β-Actin was determined using Taqman Gene Expression Assays (Life Technologies) according to the manufacturer’s instructions. Total human brain cDNA was used as a positive control for dopamine receptor amplification. Results were represented as relative expression of each gene normalized to β-Actin as a housekeeping gene using 2 ^–ΔCt^ (arbitrary units). The ∆Ct value was determined by subtracting the average Ct of the housekeeping gene from the average Ct of the target gene. Expression of mRNA for each dopamine receptor was compared between Day 0 and Day 3 monocytes and for data points with normal distribution, statistical significance was determined by paired two-tailed Student’s t test. For data points that did not distribute normally, statistical significance was determined by two-tailed Wilcoxon matched-pairs signed rank test.

### Dopamine Receptor Staining by Flow Cytometry

Day 0 and Day 3 monocytes (1x10^6^) were stained with antibodies to D1R (Catalog number 324390), D3R (Catalog number 324402), D5R (Catalog number 324408) (1:5 dilution) (Calbiochem, Billerica, MA), D2R (Catalog numbers sc-9113 and sc-5303) (1:10 dilution), D4R (Catalog number sc-31480) (1:10 dilution) (Santa Cruz Biotechnology, Santa Cruz, CA) or negative control rabbit serum, rabbit IgG, mouse IgG2a (Sigma-Aldrich, St. Louis, MO) or goat IgG (Santa Cruz Biotechnology) in 50 μL on ice for 30 min. The dopamine receptor antibodies are specific for extracellular regions of each receptor and were titered to determine optimal staining conditions for monocytes. After two washes, cells were incubated with PE-conjugated anti-rabbit, mouse or goat IgG (1:5 or 1:10 dilution) (Sigma-Aldrich) on ice in the dark for 30 min., washed twice, and fixed in 2% paraformaldehyde. Fluorescence intensity was acquired using a BD FACSCanto-II flow cytometer (BD Biosciences, San Jose, CA). Any background reactivity with the appropriate isotype matched negative control antibody was subtracted from the dopamine receptor signal to determine the mean fluorescence intensity for each dopamine receptor.

### Western Blot Analysis

Day 3 monocytes were incubated in RPMI 1640 media without serum for 45 minutes at 37°C, 5% CO_2_. Dopamine (Sigma-Aldrich), freshly resuspended in ddH_2_O, was added to a final concentration of 100 nM, 500 nM, or 1 μM for 5 or 15 minutes and the cells were then lysed with lysis buffer (Cell Signaling Technology, Danvers, MA) containing protease and phosphatase inhibitors (Sigma-Aldrich). As a negative control, lysates were prepared from cells treated with diluent. Protein concentrations were determined by Bradford assay and equal amounts of protein were separated by electrophoresis using 10% polyacrylamide gels and transferred to nitrocellulose membranes (GE Healthcare Life Sciences). Membranes were blocked for 1 hour at room temperature with 5% nonfat dry milk and 3% BSA in TBST (Tris buffered saline, with 0.1% Tween). Blots were probed with antibody against phospho-Erk1/2 (Catalog number 9106, Cell Signaling Technology) (1:2000 dilution) overnight at 4°C, washed with TBST, and probed with anti-mouse IgG-HRP secondary antibody (Catalog number 7076, Cell Signaling Technology) (1:2000 dilution) for 1 hour at room temperature. Signal was detected using Western Lightning Plus-ECL (Perkin Elmer, Waltham, MA). Blots were stripped using Restore Plus Western Blot Stripping Buffer (Thermo Scientific), and reprobed with antibody against total Erk1/2 (Catalog number 9102, Cell Signaling Technology) (1:1000 dilution) and anti-rabbit IgG-HRP secondary antibody (Catalog number 7074, Cell Signaling Technology) (1:2000 dilution). Data were quantified by densitometry using UN-SCAN-IT software (Silk Scientific, Orem, Utah). Phosphorylated Erk1/2 was normalized to total Erk1/2 protein and data were reported as percent increase in Erk1/2 phosphorylation with dopamine treatment relative to control, which was set to 0%.

### Migration Assay

Migration assays were performed using a NeuroProbe 48 well Micro Chemotaxis Chamber (Neuroprobe, Gaithersburg, MD). Chemotaxis media (RPMI 1640 supplemented with 2% FBS) alone or containing dopamine (100 nM, 500 nM, or 1 μM), SKF38393 (1 nM, 10 nM, or 100 nM), a D1-like dopamine receptor agonist, or CXCL12 (1 ng/mL), a positive control for gradient dependent migration, was placed in wells in the bottom chamber of the apparatus. Dopamine and SKF38393 were resuspended in ddH_2_O and CXCL12 was resuspended in PBS containing 0.1% BSA. A polycarbonate filter containing 5 μm pores was coated with 0.2% gelatin, dried, and placed between the bottom and top chambers. Day 3 monocytes (1x10^6^ or 3x10^6^) in chemotaxis media were added to the wells in the top chamber and allowed to migrate for 1 hour at 37°C. The membrane was then removed and the cells that had migrated through the membrane and bound to its underside were fixed and stained with Diff-Quik Stain Set (Siemens, Munich, Germany). Migration was quantified by densitometry using UN-SCAN-IT software (Silk Scientific). Transmigration in response to dopamine, SKF38393 or CXCL12 was reported as percent increase over the baseline migration, which was set to 0%.

Checkerboard assays were also performed using the Micro Chemotaxis Chamber to determine whether cell migration was gradient dependent. To establish a positive gradient, dopamine, SKF38393, or CXCL12 was placed in the wells in the bottom chamber, and Day 3 monocytes (3x10^6^ cells) in media were added to the top chamber. A negative gradient was established by placing media in the bottom chamber and adding Day 3 monocytes in media containing dopamine, SKF38393, or CXCL12 to the wells in the top chamber. A null gradient was established by adding dopamine, SKF38393, or CXCL12 to the media in the bottom chamber and to the cells added to the wells in the top chamber. Cells were allowed to migrate for 1 hour at 37°C, collected, and quantified as described above.

### Live Cell Imaging and Image Analysis

Day 3 monocytes (1.6 mL at 1.66x10^5^ cells/mL) were placed in a 35mm Easy Grip Tissue Culture Dish (BD Biosciences). Dopamine was immediately added to the dish to a final concentration of 1 μM and mixed by pipetting. An equal volume of media was added to a separate dish of cells as the negative control. Cells on the surface of the dish were imaged for 1 hour at 15 second intervals on a Zeiss Observer microscope using Axiovision Software (Carl Zeiss Microscopy, Oberkochen, Germany). Cells were viable for the duration of the experiment, as evidenced by their movement throughout the time course. The number of cells that settled on the surface of the tissue culture dish at each time point were counted using Adobe Photoshop CS4 (Adobe, San Jose, CA), and the rate of settling was calculated using linear regression.

### Spreading and Adhesion assays

To quantify adhesion dependent cell spreading, glass coverslips were placed in 24 well plates. Day 3 monocytes (1x10^5^ cells) in RPMI 1640 with 10% FBS were added to each well and allowed to settle for 15 minutes on ice. Dopamine was added to a final concentration of 1 μM or an equivalent volume of diluent was used as a negative control. Cells were warmed to 37°C for 5, 8, 10, 15, 20, and 30 minutes, and the coverslips were then placed in 2% paraformaldehyde. Fixed cells on each coverslip were permeabilized with 0.1% Triton X-100 and stained with Texas Red phalloidin and DAPI (Life Technologies) to label actin and nuclei, respectively. Stained cells were visualized by fluorescence microscopy on a Zeiss Observer microscope using Axiovision Software (Carl Zeiss Microscopy). The number of cells, as indicated by DAPI staining, and the total area of cells, as indicated by actin staining, in six separate fields were measured using Volocity 3D Image Analysis Software (Perkin Elmer). The mean area per cell was calculated by dividing the total area by the number of nuclei on each coverslip. Data were represented as the percent change in mean area of dopamine treated cells relative to control cells, which was set to 0%.

To quantify cell adhesion, glass coverslips were placed in 24 well plates. Day 3 monocytes (1.5x10^5^ cells) in RPMI 1640 with 2% FBS were added to each well. Dopamine (1 μM final concentration) or diluent were immediately added to each well and the plates were incubated at 37°C, 5% CO_2_ for 8, 10, 15, 30, 45 and 60 minutes. Following incubation, each well was washed 3 times with warm PBS and adherent cells on each coverslip were fixed with 2% paraformaldehyde. Cells were stained for actin and DAPI and visualized by fluorescence microscopy on a Zeiss Observer microscope using Axiovision Software (Carl Zeiss Microscopy). For each coverslip, the number of cells in fourteen separate fields was quantified using Volocity 3D Image Analysis software (Perkin Elmer).

### Viability assay

Day 3 monocytes (1x10^5^ cells) in RPMI 1640 with 10% FBS were added to 35mm glass bottomed MatTek dishes (MatTek Corporation, Ashland, MA) in the presence of 1 μM dopamine or diluent for 30 minutes at 37°C. Cell viability was then determined using the LIVE/DEAD Viability/Cytotoxicity Kit (Life Technologies) according to the manufacturer’s instructions. Viability was evaluated using fluorescence microscopy to visualize cells, with live cells fluorescing green and dead cells fluorescing red.

### Statistical Analysis

Statistical analyses were performed using Prism 6.02 (GraphPad Software, Inc., San Diego, CA). Two-tailed Wilcoxon matched-pairs signed rank tests or paired Student’s t tests were used to determine statistical significance, with p < 0.05 considered to be significant.

## Results

### Day 3 monocytes express dopamine receptors

Studies from other groups examining dopamine receptors on monocytes used freshly isolated cells [52,53], which are primarily CD14^+^CD16^-^. For this study, we focused on mature/activated CD14^+^CD16^+^ monocytes, as these are the cells that preferentially cross the blood brain barrier and are key mediators of HIV associated neuropathogenesis [13,21,23,25–27,54]. Because CD14^+^CD16^+^ monocytes comprise only 5–10% of peripheral blood monocytes in healthy individuals, our laboratory developed a culture system to enrich for the more mature CD14^+^CD16^+^ subpopulation to obtain sufficient cell numbers for further study. Briefly, CD14^+^ monocytes were isolated from PBMC and cultured non-adherently in Teflon flasks for three days in the presence of M-CSF, which resulted in cell cultures containing 65–95% CD14^+^CD16^+^ monocytes. Freshly isolated monocytes are termed “Day 0” and matured/activated cell cultures are called “Day 3” [21,23]. Throughout the text we refer to these mature/activated monocytes as “mature” monocytes for simplicity.

**Fig 1:**
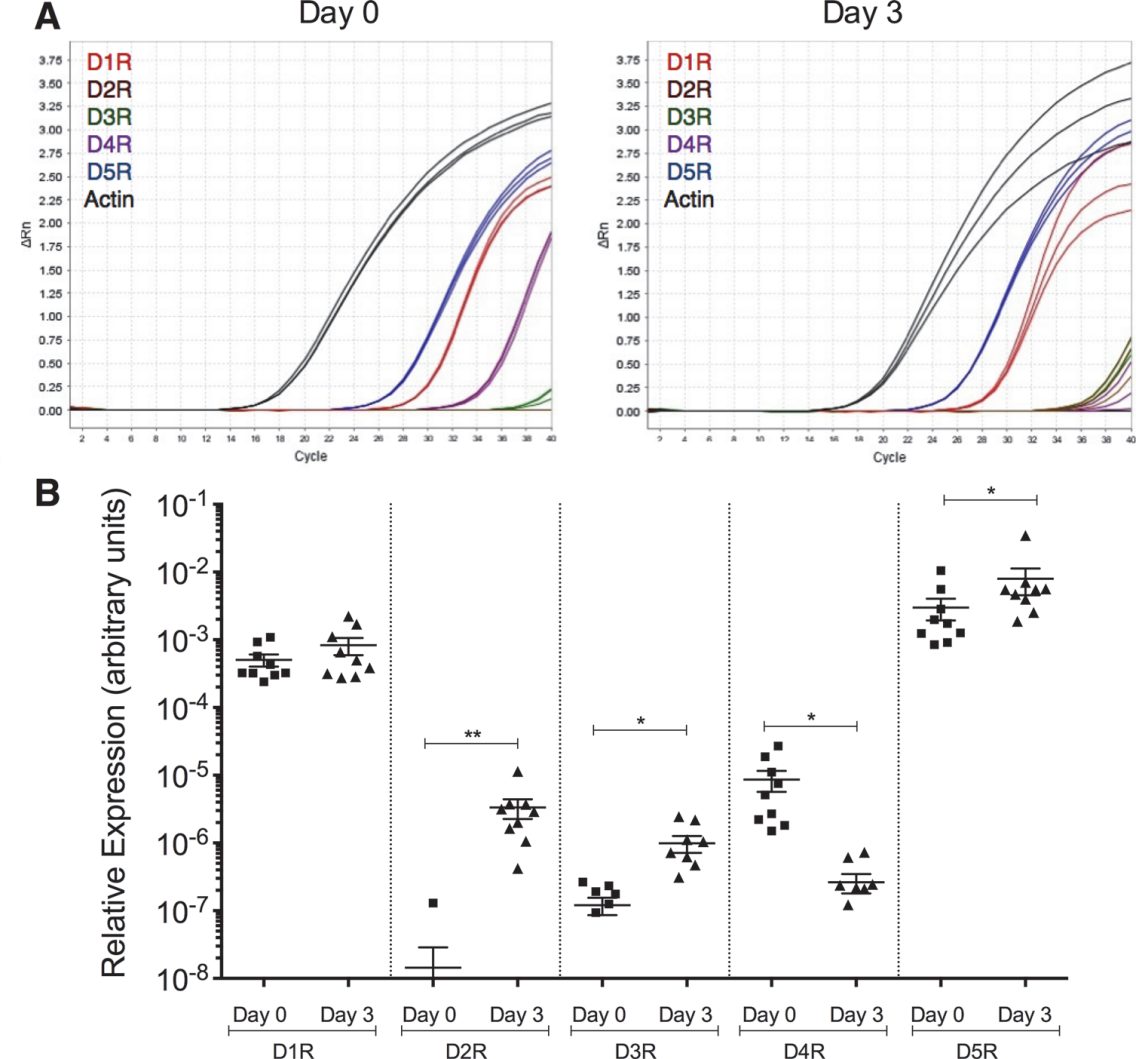
Dopamine receptor mRNA expression changes with monocyte maturation. Day 0 and Day 3 monocytes were analyzed by qRT-PCR for dopamine receptor mRNA expression. (A) Amplification plots from a representative donor showing mRNA expression of D1R, D2R, D3R, D4R, D5R and actin in Day 0 and Day 3 monocytes. (B) Relative expression of dopamine receptors normalized to actin for all donors tested. Data points for D1R, D3R and D4R distributed normally and were analyzed by two-tailed paired Student’s t-test for significance. Data points for D2R and D5R did not have a normal distribution and were analyzed by two-tailed Wilcoxon matched-pairs signed rank test for significance (*p<0.05, **p<0.01, N = 9).

To determine the effect of monocyte maturation on dopamine receptor expression, we analyzed dopamine receptor mRNA expression in Day 0 and Day 3 monocytes by qRT-PCR (Fig. 1A,B). Representative amplification plots from a single donor show that Day 0 monocytes expressed mRNA for all dopamine receptors except D2R, while Day 3 monocytes expressed mRNA for all five dopamine receptors (Fig. 1A). In all donors tested, expression of D2R (**p < 0.01, N = 9), D3R (*p < 0.05, N = 9), and D5R (*p < 0.05, N = 9) mRNA significantly increased with maturation, while D4R significantly decreased (*p < 0.05, N = 9) (Fig. 1B). D1R mRNA expression also trended toward an increase with maturation, but the difference was not statistically significant (N = 9).

**Fig 2:**
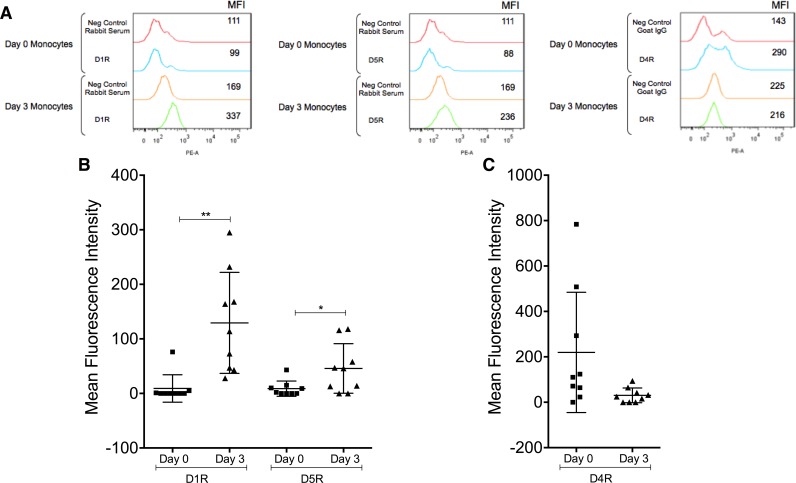
Dopamine receptor surface protein expression changes with monocyte maturation. Day 0 and Day 3 monocytes were analyzed by flow cytometry for surface expression of D1R, D5R, and D4R. (A) Representative histograms from a single donor showing increased surface expression of D1R and D5R, and decreased expression of D4R with maturation. Analysis of cell cultures from all donors shows surface expression of (B) D1-like dopamine receptors, D1R and D5R, increasing with maturation (*p<0.05, **p<0.01, N = 9) and (C) D4R surface expression trending towards a decrease with maturation (p = 0.07, N = 9) (two-tailed Wilcoxon matched-pairs signed rank test).

We next examined cell surface dopamine receptor protein by flow cytometry (Fig. 2). Fig. 2A shows representative histograms of D1R, D4R, and D5R expression in a single donor. Mean fluorescence intensity of dopamine receptor signal above that obtained with the appropriate negative control antibodies shows increased expression of D1R and D5R and decreased D4R on Day 3 monocytes when compared to Day 0 monocytes. Data compiled from all experiments show that surface D1R and D5R protein on monocytes significantly increased with maturation in all donors tested (**p<0.01, *p < 0.05, N = 9)(Fig. 2B). D4R protein trended toward a decrease, but the difference between Day 0 and Day 3 monocytes was not statistically significant because of the variability among primary cells from individual donors (p = 0.07, N = 9)(Fig. 2C). Differences in dopamine receptor expression with monocyte maturation support other data from our laboratory demonstrating that mature CD14^+^CD16^+^ monocytes are functionally distinct from less mature CD14^+^CD16^-^ monocytes [21,23]. The presence of dopamine receptors on CD14^+^CD16^+^ monocytes suggests that these cells will respond to dopamine.

We were unable to detect surface D2R and D3R by flow cytometry because very few antibodies target extracellular epitopes of these receptors and staining with several of those that do showed no signal above the appropriate isotype matched negative control antibodies. Other groups showed surface expression of all dopamine receptors on human immune cells by flow cytometry [52,55,56]. However, some of these studies used secondary antibody only in the absence of negative control antibody or did not use the appropriate negative control antibody, which may account for differences in detection of these receptors by flow cytometry.

### Dopamine receptors expressed by Day 3 monocytes are functional

**Fig 3:**
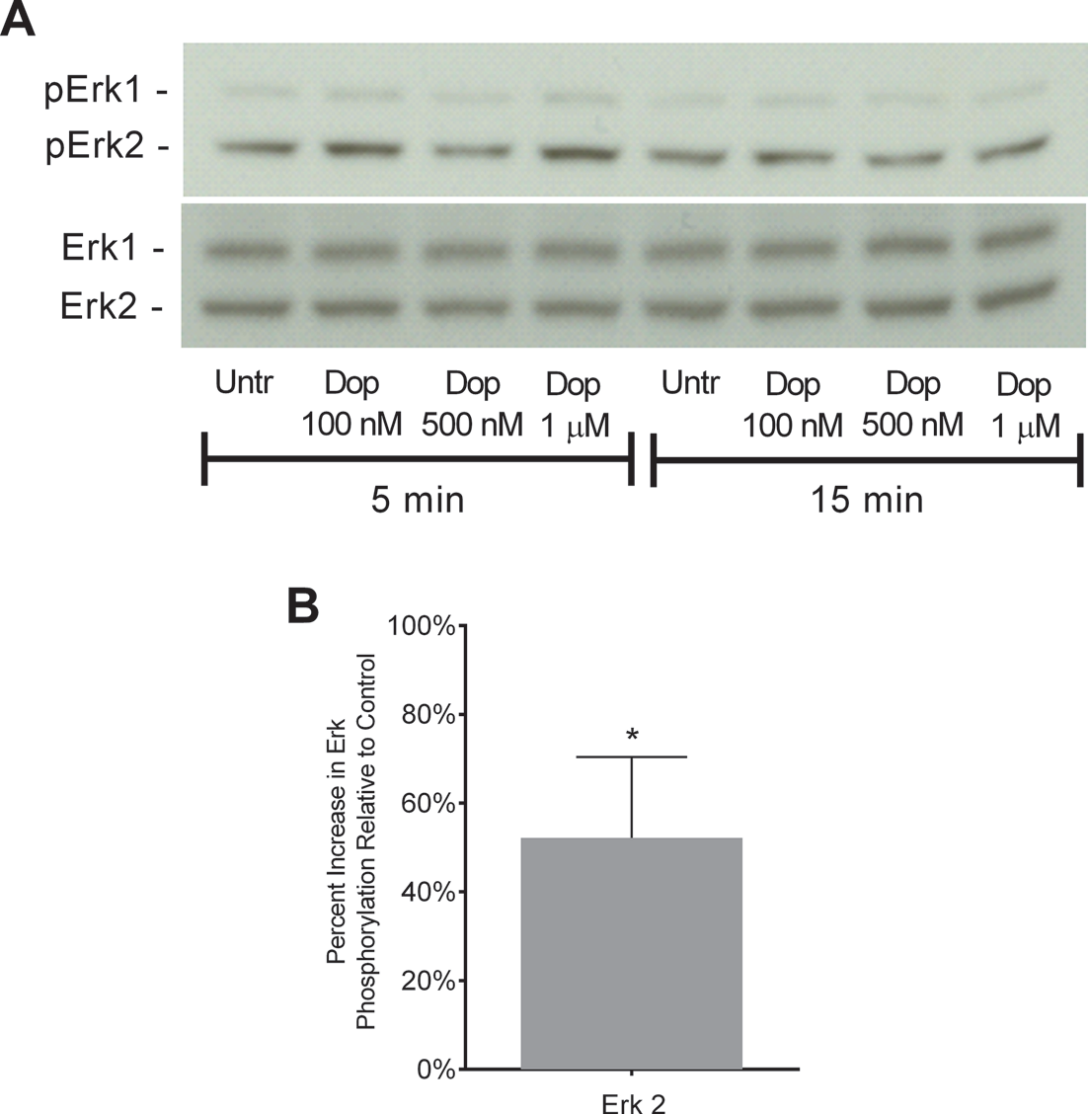
Dopamine receptor activation increases Erk2 phosphorylation. Day 3 monocytes were treated with dopamine (100 nM, 500 nM, 1 μM) for 5 or 15 minutes, and protein lysates were analyzed by Western blot. (A) Representative blot showing changes in Erk1/2 phosphorylation in response to dopamine treatment. Total Erk1/2 was used as a loading control. (B) Quantification of changes in Erk2 phosphorylation normalized to total Erk2, shown as maximal percent change at 5 or 15 minutes relative to untreated cells, which is set to 0% (*p<0.05, N = 6) (two-tailed Wilcoxon matched-pairs signed rank test).

Erk1/2 phosphorylation is part of signaling pathways that mediate many cell processes, including cell migration [57] and dopamine increases Erk1/2 phosphorylation in neurons and macrophages [48,58,59]. To determine whether dopamine receptors expressed on Day 3 monocytes are functional, we examined the phosphorylation of Erk1/2 in these cells in response to dopamine. Day 3 monocytes were treated with dopamine (100 nM, 500 nM, or 1 μM) for 5 or 15 minutes, and protein lysates from these cells were analyzed by Western blot. Fig. 3A shows a representative Western blot from a single donor in which the greatest increase in Erk2 phosphorylation was induced by 1 μM dopamine treatment for 5 minutes. Due to the variability inherent in primary cells, the time at which 1 μM dopamine induced the maximal increase in Erk2 phosphorylation varied between 5 and 15 minutes among individual donors. Densitometric analysis of data from six independent experiments quantified phospho-Erk2 normalized to total Erk2, which was used a loading control. The results showed that treatment of Day 3 monocytes with 1 μM dopamine maximally increased Erk2 phosphorylation after 5 or 15 minutes by 52% over baseline (Fig. 3B) (*p < 0.05, N = 6). These data demonstrate that dopamine receptors on Day 3 monocytes are functional, as evidenced by dopamine-induced phosphorylation of Erk2.

### Dopamine and D1-like DR activation increase Day 3 monocyte migration

**Fig 4:**
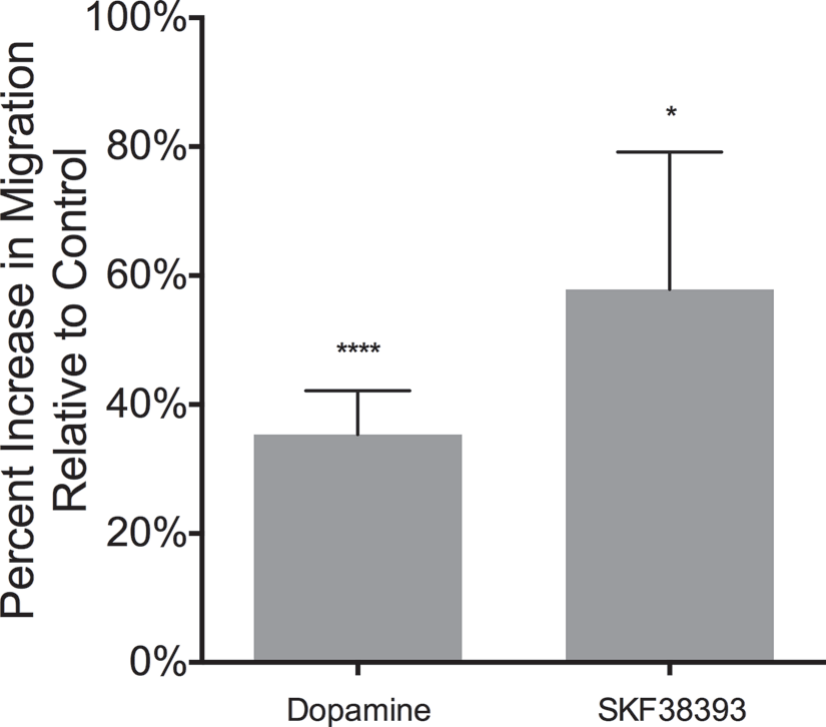
Dopamine and D1-like dopamine receptor activation increase migration of Day 3 monocytes. Day 3 monocytes were added to the top wells of a microchemotaxis chamber. Dopamine (100 nM, 500 nM, 1 μM) (N = 19) or D1-like receptor agonist SKF38393 (1 nM, 10 nM, 100 nM) (N = 6) was added to the bottom chamber. Cells were allowed to migrate for 1 hour at 37°C through a polycarbonate membrane. Cells that migrated and adhered to the underside of the membrane were fixed, stained, and quantified by densitometry. Migration is shown as the maximal percent increase with 100 nM, 500 nM or 1 μM dopamine, relative to baseline migration, which is set to 0% (*p<0.05, ****p<0.0001) (two-tailed Wilcoxon matched-pairs signed rank test).

In drug abusers, CD14^+^CD16^+^ monocytes can be exposed to increased extracellular dopamine once they have entered the CNS in response to chemokines. Dopamine has been shown to induce migration of rodent microglial cells [60] and another study suggested that dopamine may be chemotactic for resting human T cells [53]. However, the effects of dopamine on monocyte migration are unknown. To study the effects of dopamine specifically on CD14^+^CD16^+^ monocyte migration, we performed a migration assay using a 48 well chemotaxis chamber. Dopamine (100 nM, 500 nM, or 1 μM) or media alone was added to the wells in the bottom chamber and Day 3 monocytes were placed in the top chamber. After 1 hour at 37°C, the cells that had migrated through a polycarbonate filter and adhered to the underside of the filter were fixed, stained, and analyzed by densitometry. Dopamine at 100 nM, 500 nM or 1 μM, significantly increased migration of Day 3 monocytes by 35% (****p < 0.0001, N = 19), as compared to media alone, which was set to 0% (Fig. 4). The concentration of dopamine that elicited maximal migration varied among donors, likely due to the inherent variability in primary cells.

To determine whether dopamine mediated migration was a result of activation, at least in part, of D1-like dopamine receptors, we used the D1-like dopamine receptor agonist SKF38393 (1 nM, 10 nM, or 100 nM) in migration assays. Due to the variability inherent in using primary cells, the concentration of SKF38393 that induced maximal migration varied among donors. This agonist significantly increased Day 3 monocyte migration by 57% over baseline migration (Fig. 4) (*p < 0.05, N = 6). These data indicate that activation of D1-like dopamine receptors mediates, at least in part, dopamine-induced migration of CD14^+^CD16^+^ monocytes.

Although we could not detect surface D2R or D3R on Day 3 monocytes by flow cytometry, we did demonstrate D2R and D3R mRNA expression in these cells. Therefore, we also performed migration assays using quinpirole, a D2-like dopamine receptor agonist. Experiments using quinpirole yielded highly variable and non-significant results (data not shown). The role of D2-like dopamine receptors in CD14^+^CD16^+^ monocyte migration will be examined further in future studies.

### Dopamine induced increase in migration is not gradient dependent

**Fig 5:**
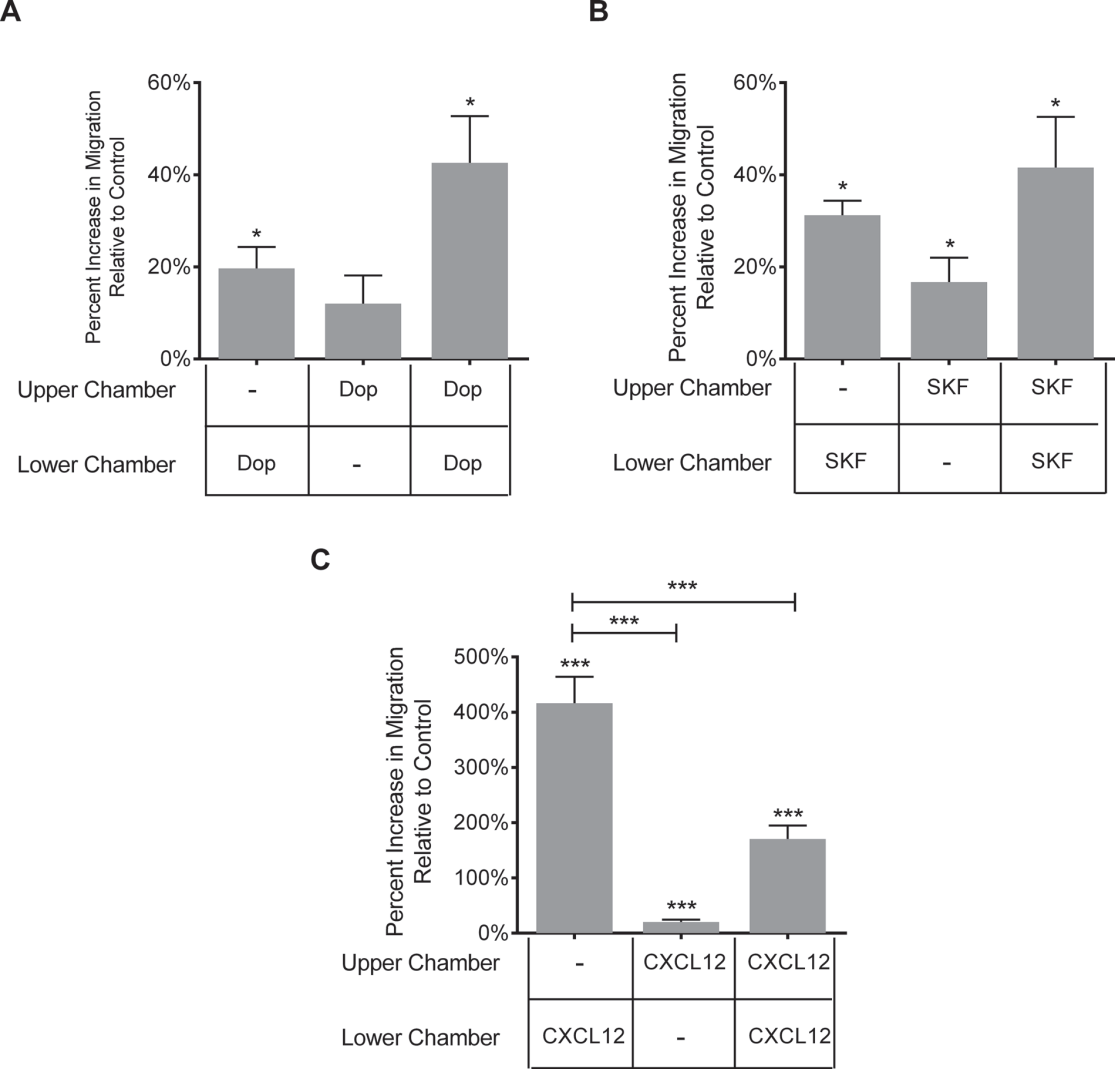
Dopamine induced migration is not gradient dependent. A checkerboard assay was performed by generating positive, negative, and null gradients using a microchemotaxis chamber. (A) Dopamine (100 nM, 500 nM or 1 μM) and (B) D1-like dopamine receptor agonist SKF38393 (1, 10 or 100 nM) increased migration of Day 3 monocytes in a gradient independent manner, as evidenced by increased transmigration when added to the top chamber as well as to both the top and the bottom chamber (N = 6). (C) CXCL12 (1 ng/ml), used as a positive control for chemotaxis, induced maximal migration when present only in the bottom chamber, indicative of gradient dependent transmigration. Migration is shown as maximal percent increase relative to baseline migration, which is set to 0% (*p<0.05, ***p<0.001, N = 12) (two-tailed Wilcoxon matched-pairs signed rank test).

Cell movement can be either directional or random. Chemotaxis is directional movement that is gradient dependent. In contrast, chemokinesis is random cell movement that does not require a gradient. To determine whether the increased migration of Day 3 monocytes in response to dopamine or D1-like DR activation was gradient dependent, we performed a checkerboard analysis, as described in Materials and Methods, in which the cells were allowed to migrate in response to positive, negative, and null gradients. When dopamine was present only in the bottom chamber or in both the top and bottom chambers, Day 3 monocyte migration significantly increased, as compared to media (Fig. 5A)(*p < 0.05, N = 6). SKF38393 significantly increased Day 3 monocyte migration when present only in the top chamber, only in the bottom chamber, or in both the top and bottom chambers (Fig. 5B)(*p < 0.05, N = 6). In contrast, CXCL12, used as a positive control for chemotaxis, induced maximal migration of Day 3 monocytes when in the bottom chamber, in response to a positive chemokine gradient (Fig. 5C, ***p < 0.001, N = 12). A negative or null gradient induced significantly less migration, indicating a chemotactic response to CXCL12 (***p < 0.001, N = 12). This assay showed that dopamine-induced migration of Day 3 monocytes is due to chemokinesis and suggests that the increased migration may be due to faster cell movement and/or increased adhesion to the polycarbonate filter.

### Dopamine increases accumulation of Day 3 monocytes

**Fig 6:**
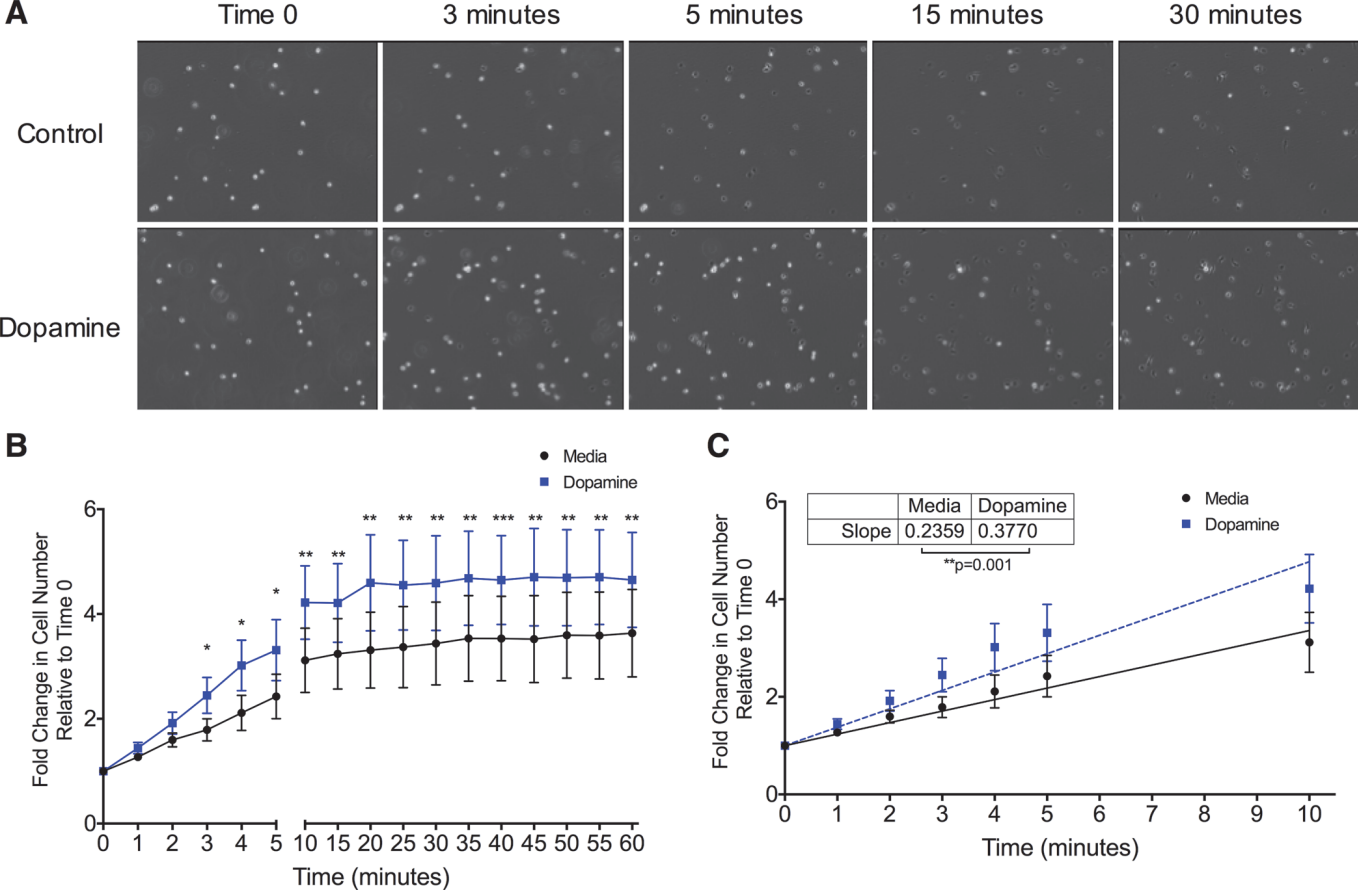
Dopamine increases the accumulation of Day 3 monocytes and their rate of settling. Day 3 monocytes were added to tissue culture dishes in the presence of dopamine (1 μM) or media, and the surface of each dish was imaged for 1 hour at 15 second intervals to record cells as they settled. Brightness was increased equally for all images. (A) Images from a representative experiment showing more monocytes settling to the surface of tissue culture dishes in the presence of dopamine at multiple time points when compared to cells without dopamine (control). (B) The addition of dopamine resulted in increased numbers of settled cells, as shown by fold change relative to Time 0, which is set to 1 (*p<0.05, **p<0.01, ***p<0.001 relative to media, N = 6) (two-tailed paired Student’s t-test). (C) Dopamine significantly increased the rate at which Day 3 monocytes settled to the surface of tissue culture dishes (**p = 0.001, N = 6) (two-tailed paired Student’s t-test).

To examine the effect of dopamine on monocyte motility and adhesion, we performed live cell imaging to observe Day 3 monocytes as they settled from suspension onto the surface of a tissue culture dish in the presence or absence of dopamine (see S1 Fig. for design of experiment). Fig. 6A shows selected time points from a representative experiment in which monocytes in the presence of either 1μM dopamine or media were allowed to settle for the indicated times. To quantify the settling process, cells on the surface of the tissue culture dish at each time point were counted and normalized to the number of cells on the tissue culture surface at Time 0. Data from independent experiments are shown as fold change at each time point relative to Time 0, which was set to 1, for each treatment group (Fig. 6B). Day 3 monocytes in the presence of dopamine showed significantly increased accumulation on the plate surface as compared to control at each time point, starting at 3 minutes after the addition of dopamine (Fig. 6A,B and S1 and S2 Videos) (*p < 0.05, **p<0.01, N = 6). The rate at which the dopamine treated cells accumulated was significantly faster than the rate of accumulation of control cells, as determined using linear regression to calculate the slope of the fit line (Fig. 6C) (***p = 0.001, N = 6). With dopamine or media only, the rate plateaued by 15 minutes. These data suggest that dopamine increases the accumulation and kinetics of adherence of Day 3 monocytes as they settle and adhere to the surface of a tissue culture dish.

### Dopamine increases the area and number of adhering Day 3 monocytes

**Fig 7:**
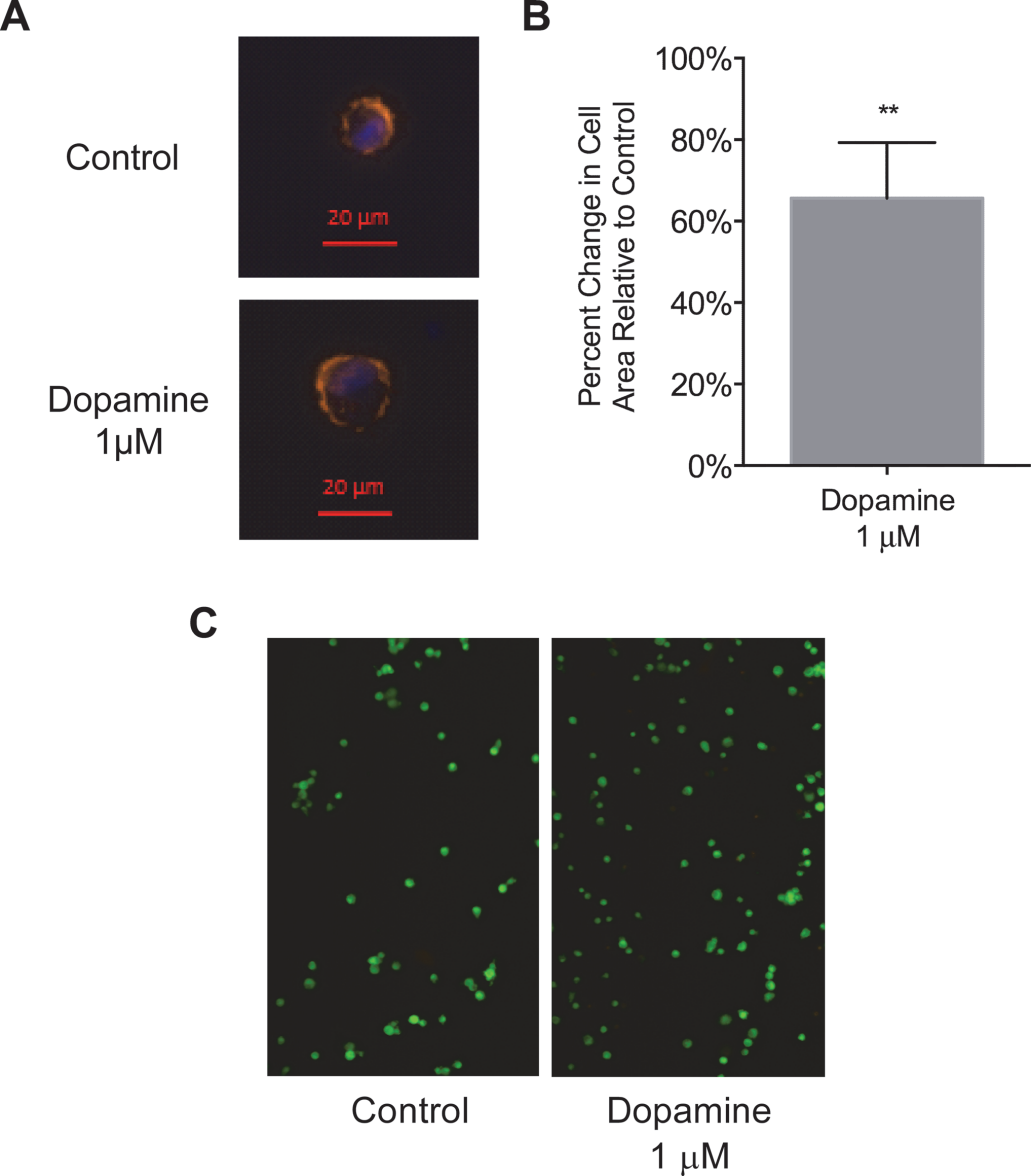
Dopamine increases the area of Day 3 monocytes during the early stages of adhesion. (A) Representative images from a single donor showing increased area of a Day 3 monocyte in the presence of 1 μM dopamine after 10 minutes of adhesion as compared to a non-dopamine treated control cell. (B) Quantification of percent change in cell area as compared to control, which is set to 0%. Data are shown as the maximal increase in area per cell, relative to control. The time of maximal increase varied between 8, 10, and 15 minutes among independent donors due to inherent variability in primary cells (**p<0.01, N = 5) (two-tailed paired Student’s t-test). (C) Viability assay of Day 3 monocytes after incubation in media (control) or 1 μM dopamine for 30 min. Live cells exhibit green fluorescence and dead cells exhibit red fluorescence.

The live cell imaging data suggest that dopamine increases the accumulation and adhesion of CD14^+^CD16^+^ monocytes. To evaluate the effects of dopamine on the initial stages of monocyte adhesion, we quantified adhesion dependent cell spreading of Day 3 monocytes in the presence or absence of dopamine. Day 3 monocytes were added to glass coverslips in 24 well tissue culture plates and allowed to settle on ice. The low temperature prevents adhesion to the surface as the monocytes, which are in suspension, settle onto the coverslips. Dopamine was added to a final concentration of 1 μM, and the cells were warmed to 37°C. An equivalent volume of diluent was used as a control. At each time point after the addition of dopamine (5, 8, 10, 15, 20 and 30 minutes), the coverslips were placed in 2% paraformaldehyde. Fixed adherent cells on each coverslip were stained with Texas Red phalloidin and DAPI to visualize the actin cytoskeleton and nuclei, respectively. Six fields from each coverslip were visualized using fluorescence microscopy. Actin staining was used to measure the area of all the cells on the coverslip, and the number of nuclei, as indicated by DAPI staining, determined the cell count. The mean area per cell was calculated by dividing the total area of the cells by the number of nuclei on each coverslip and the data were represented as the percent change in the area of dopamine treated cells relative to control cells, which was set to 0%. The 5 minute time point had very few cells adhered to the coverslips and therefore was not included in our analysis. Because of the inherent variability in primary cells, the time of maximal increased cell area induced by dopamine varied among donors. In a representative experiment, the area of a Day 3 monocyte after 10 minutes of adhesion was increased by dopamine when compared to an untreated (control) cell (Fig. 7A). Data from independent experiments showed that dopamine significantly increased adhesion dependent Day 3 monocyte cell spreading, as compared to media alone, at early time points (8, 10, or 15 minutes) (Fig. 7B)(**p < 0.01, N = 5). This effect was lost by 20 minutes (data not shown). We also performed a viability assay of Day 3 monocytes after a 30 minute incubation with dopamine and determined that the cells were viable and therefore treatment with 1μM dopamine was not toxic (Fig. 7C). These data indicate that dopamine increases the spreading of Day 3 monocytes during the early stages of adhesion.

**Fig 8:**
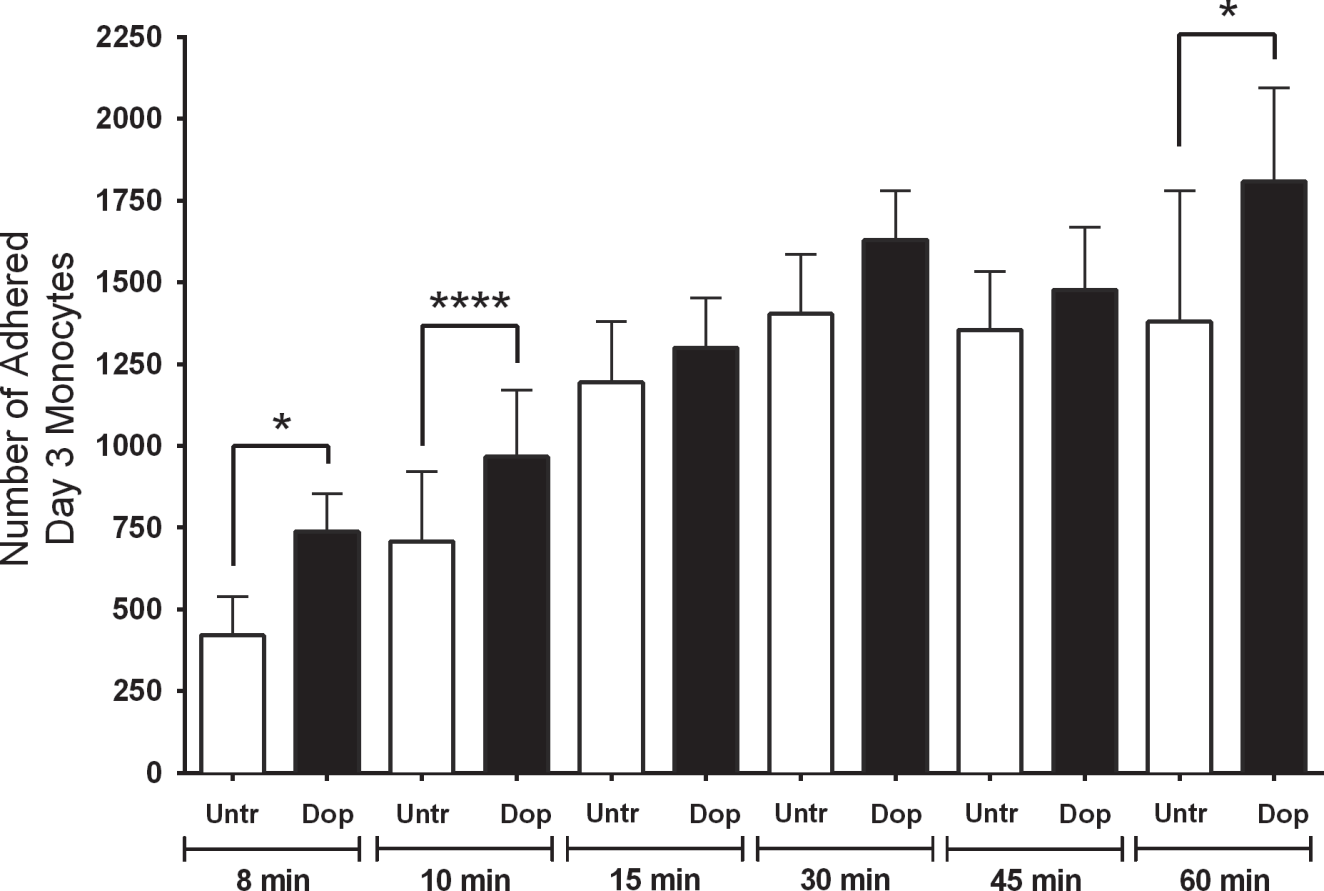
Dopamine increases the adhesion of Day 3 monocytes. (A) Dopamine increases the adhesion of Day 3 monocytes after 8, 10 and 60 minutes. Data are shown as the number of adherent Day 3 monocytes in the presence of 1 μM dopamine or diluent (untreated) after multiple time points (*p<0.05, ***p<0.001, N = 5 for 8 and 10 min and N = 6 for 15, 30, 45 and 60 min) (two-tailed paired Student’s t-test).

Adhesion assays were performed to determine whether dopamine increased overall CD14^+^CD16^+^ monocyte adhesion. After the addition of Day 3 monocytes and 1 μM dopamine or diluent to glass coverslips in 24 well plates, the cells were allowed to adhere at 37°C, 5% CO_2_ for 8, 10, 15, 30, 45 or 60 minutes. Following incubation, coverslips in each well were washed with PBS to detach non-adherent cells and after fixation, adherent cells on each coverslip were stained with Texas Red phalloidin and DAPI. Fourteen fields from each coverslip were visualized using fluorescence microscopy and the number of adherent cells was quantified by counting cell nuclei. Data from five independent experiments showed that dopamine increased the number of adherent Day 3 monocytes when compared to diluent treated control cells (untreated) at the 8 and 10 min time points (Fig. 8) (*p<0.05, ***p<0.001, N = 5). At later time points adhesion was not significantly increased with dopamine except for the 60 minute time point (*p<0.05, N = 6).

## Discussion

**Fig 9:**
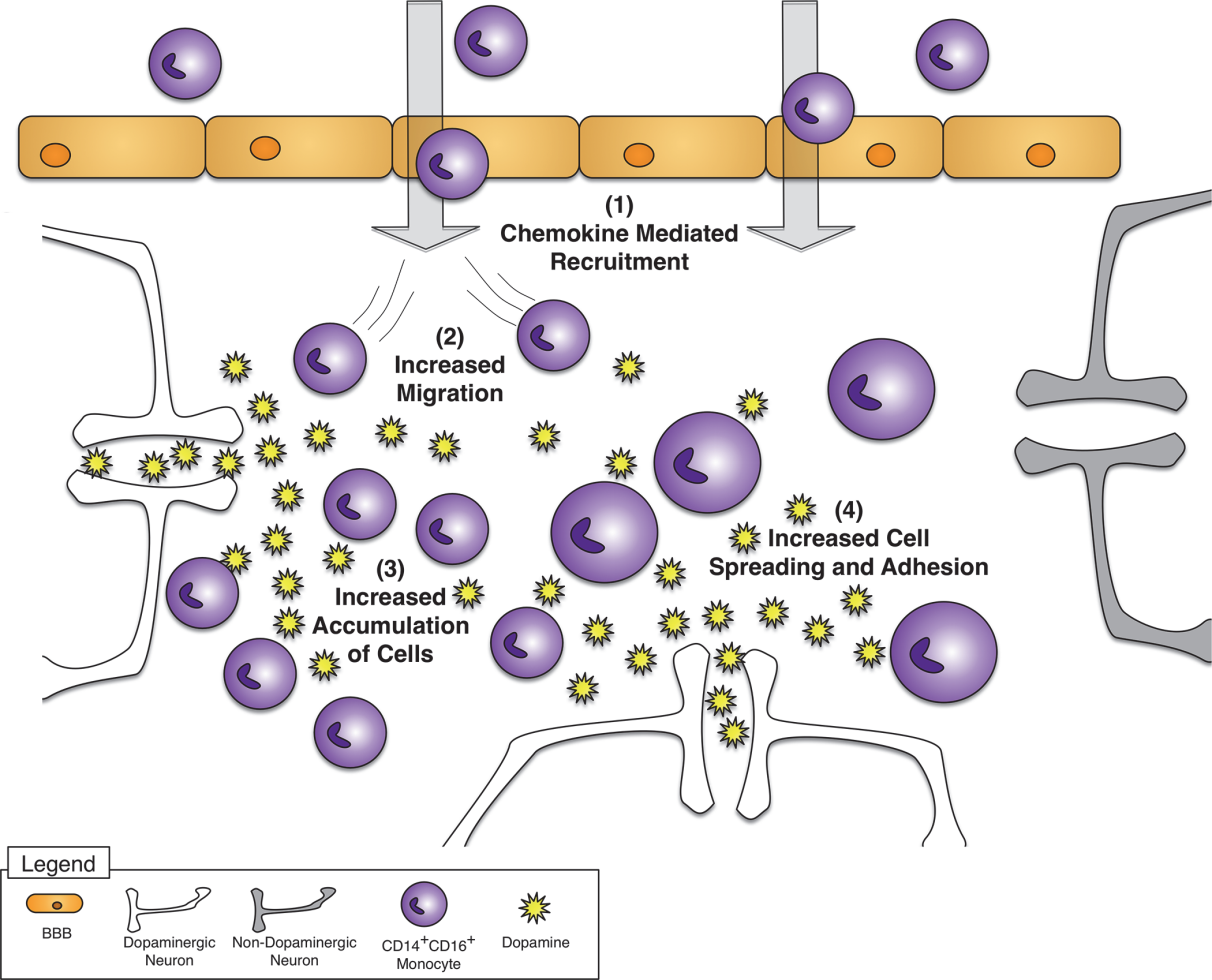
Dopamine increases CD14^+^CD16^+^ monocyte migration and adhesion. (1) CD14^+^CD16^+^ monocytes enter the CNS in response to increased chemokines and then encounter increased extracellular dopamine due to drug abuse. (2) CD14^+^CD16^+^ monocytes migrate more in response to dopamine. Dopamine increases the (3) accumulation and (4) spreading and adhesion of CD14^+^CD16^+^ monocytes. This may alter the distribution of CD14^+^CD16^+^ monocytes in the CNS, leading to accumulation of these cells in dopaminergic brain regions. Increased CD14^+^CD16^+^ monocyte accumulation may contribute to neuroinflammation and neuronal damage.

Monocytes, including CD14^+^CD16^+^ monocytes, enter the brains of HIV infected individuals in response to increased chemokines [8,9,11–14,21]. Drug abuse increases extracellular CNS dopamine [32–37], which can affect these cells once they have crossed the BBB. In this study, we found that CD14^+^CD16^+^ monocytes express mRNA or surface protein of all five dopamine receptors. In addition, surface expression of the D1-like dopamine receptors, D1R and D5R, increased in CD14^+^CD16^+^ monocytes when compared to freshly isolated monocyte cultures comprised predominately of CD14^+^CD16^-^ monocytes. Dopamine increased Erk2 phosphorylation in CD14^+^CD16^+^ monocytes, indicating that these cells expressed functional dopamine receptors. To characterize the effects of dopamine on CD14^+^CD16^+^ monocyte migration and adhesion, we performed migration, settling, spreading and adhesion assays. Dopamine and the D1-like dopamine receptor agonist SKF38393 increased the migration of CD14^+^CD16^+^ monocytes, which was not dependent on a gradient. To characterize further the dopamine-induced changes in the movement of CD14^+^CD16^+^ monocytes, we performed a settling assay. In this assay, dopamine increased the number and rate at which CD14^+^CD16^+^ monocytes in suspension accumulated onto the surface of tissue culture dishes. Lastly, in spreading and adhesion assays, dopamine increased the area of CD14^+^CD16^+^ monocytes during the early stages of adhesion and the overall total number of adherent cells. These findings are summarized in Fig. 9. Taken together, our data suggest that once CD14^+^CD16^+^ monocytes transmigrate across the blood brain barrier into the brain parenchyma of HIV infected drug abusers in response to chemokines, these cells will adhere and accumulate within dopaminergic regions of the brain. This may be a mechanism by which drug abuse increases HIV associated neuroinflammation and the severity of HAND.

Neuroinflammation associated with HIV infection and HAND impacts regions of the brain that are enriched for dopaminergic neurons, particularly the frontal cortex, basal ganglia, and hippocampus [61,62]. Acute and intermittent drug abuse causes increased extracellular dopamine in these brain regions that may diffuse from the synapse into surrounding CNS tissue [51]. This increased dopamine could exacerbate HIV associated neuroinflammation by affecting monocytes that have entered the brain in response to increased chemokines. Our study showed an increase in motility and adhesion of CD14^+^CD16^+^ monocytes using dopamine concentrations ranging from 100 nM to 1 μM. While the precise concentration of dopamine in the human brain is unknown, based on rodent studies, baseline dopamine in the brain is estimated to be in the low nanomolar range [36]. Cocaine and methamphetamine use can increase dopamine levels to low micromolar concentrations [63,64]. The interaction of CD14^+^CD16^+^ monocytes with elevated extracellular dopamine due to drug abuse may increase their accumulation in dopaminergic brain regions, increasing neuroinflammation and ultimately damaging neurons and negatively impacting cognition and learning.

The mature CD14^+^CD16^+^ monocyte subpopulation is critical in HIV neuropathogenesis. This population is increased in the blood of HIV infected individuals, is highly permissive to HIV infection, and preferentially transmigrates across the BBB [7,23–25]. These cells bring HIV into the CNS early after peripheral infection, and both infected and uninfected CD14^+^CD16^+^ monocytes continue to enter the brain in response to HIV mediated increases in CCL2 and CXCL12 [2,8,11,12,21,65,66]. Dopamine does not cross the BBB [49] and will mediate its effects on these infiltrating monocytes once they have been recruited into the CNS parenchyma by chemokines. CD14^+^CD16^+^ monocytes can be exposed to increased dopamine escaping from the synapse in the CNS of a drug abuser soon after crossing the BBB [50,51], as individual neurons are estimated to be within 8–20 μm of a capillary [67]. In HIV infected individuals who abuse drugs, exposure of these accumulated monocytes, both uninfected and infected, to inflammatory mediators present within the CNS may result in their further activation and production of neurotoxic factors. Additionally, infected cells may elaborate viral proteins, which are toxic to neurons. Cumulatively, dopamine may act in concert with these mediators to contribute to chronic, low level neuroinflammation that may ultimately damage neurons and lead to neurocognitive impairments in a large number of HIV infected drug abusers.

Monocytes differentiate into macrophages after extravasation, and in addition to affecting the accumulation of CD14^+^CD16^+^ monocytes and consequently macrophages in the CNS, increased CNS dopamine may also contribute to neuropathogenesis by altering macrophage function and activating viral reservoirs within the CNS. Dopamine increases HIV replication and the number of infected cells in cultures of human monocyte derived macrophages [48]. Dopamine also increases CCL2 production by macrophages [68], which contributes to neuroinflammatory processes by activating immune cells and recruiting additional monocytes from the periphery. Norepinephrine, another catecholamine neurotransmitter, induces chemotaxis of macrophages [69,70], further underscoring that neurotransmitters may modulate the inflammatory response within the brain by altering the distribution and accumulation of immune cells.

This effect of dopamine may not be limited to drug abusers infected with HIV. Other infections associated with drug abuse include hepatitis B and C [71]. Studies show increased numbers of CD14^+^CD16^+^ monocytes in the peripheral blood of people infected with these viruses [72,73]. Monocytes enter the brain as part of normal immune surveillance; therefore, individuals with increased numbers of CD14^+^CD16^+^ monocytes in the peripheral blood will have more of these cells available to enter the CNS. There is also evidence of neuroinflammation and cognitive impairment in individuals with chronic hepatitis C (HCV) infection [74–78]. Another study examining individuals with HIV, HCV and/or methamphetamine addiction found that individuals with two or three of these risk factors performed worse on cognitive tests than individuals with fewer risk factors [79]. Therefore, increased extracellular dopamine due to drug use may also contribute to neuroinflammation in drug abusers infected with hepatitis B and C by increasing the accumulation of CD14^+^CD16^+^ monocytes within dopamine rich regions of the CNS.

Future studies are needed to evaluate the effects of dopamine on migration and adhesion of HIV infected monocytes. Our laboratory demonstrated that HIV infection increased the sensitivity of CD14^+^CD16^+^ monocytes to the chemokine CCL2, resulting in their increased migration in a chemotaxis assay, as well as increased transmigration across an in vitro BBB model, as compared to uninfected cells [21]. HIV infection may similarly alter the sensitivity of CD14^+^CD16^+^ monocytes to dopamine, possibly by altering dopamine receptor expression. Studies are ongoing to address these questions.

## Supporting Information

S1 Fig Schematic describing how live cell imaging of Day 3 monocyte settling was performed as presented in S1 and S2 Videos. (TIF)

S1 Video Accumulation and adhesion of Day 3 monocytes in media alone. Day 3 monocytes suspended in media alone were added to tissue culture plates, immediately followed by the addition of diluent, and the cells were allowed to settle for 1 hour at 37°C. Images were taken every 15 seconds. Brightness was increased equally for all videos.
(AVI)

S2 Video Accumulation and adhesion of Day 3 monocytes in 1 μM dopamine. Day 3 monocytes suspended in media were added to tissue culture plates, immediately followed by the addition of 1 μM dopamine, and the cells were allowed to settle for 1 hour at 37°C (see S1 Fig.). Images were taken every 15 seconds. Brightness was increased equally for all videos.
(AVI)
