# Supplementary figures and images for: Dopamine Increases CD14+CD16+ Monocyte Migration and Adhesion in the Context of Substance Abuse and HIV Neuropathogenesis
Source: PLoS One. 2015 Feb 3;10(2):e0117450. doi: 10.1371/journal.pone.0117450 (PMC4315499; doi:10.1371/journal.pone.0117450)

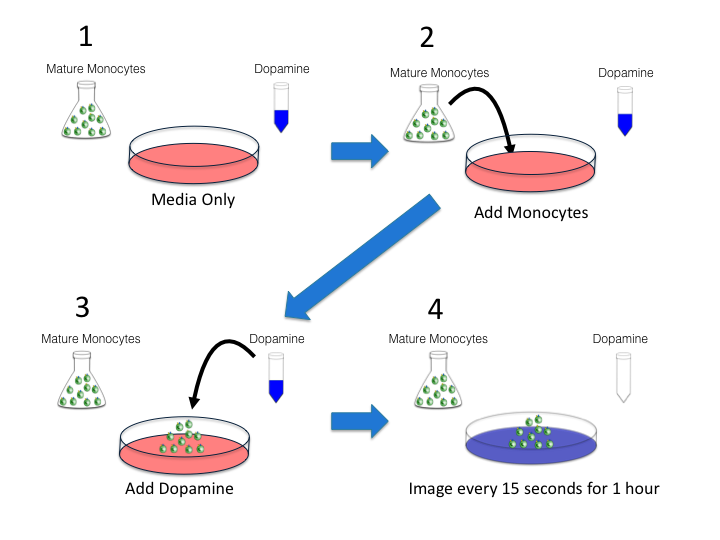

Supplement: S1 Fig — (TIF) [file pone.0117450.s001.tif]
